# Comparing Acute, High Dietary Protein and Carbohydrate Intake on Transcriptional Biomarkers, Fuel Utilisation and Exercise Performance in Trained Male Runners

**DOI:** 10.3390/nu13124391

**Published:** 2021-12-08

**Authors:** Matthew Furber, Simone Pyle, Michael Roberts, Justin Roberts

**Affiliations:** 1 Department of Psychology and Sport Sciences, School of Life and Medical Sciences, University of Hertfordshire, Hatfield AL10 9AB, UK; m.g.roberts@herts.ac.uk; 2 Food Microbial Sciences Unit, Department of Food and Nutritional Sciences, University of Reading, Reading RG6 6AP, UK; s.pyle@pgr.reading.ac.uk; 3 Cambridge Centre for Sport and Exercise Sciences, School of Psychology and Sport Science, Anglia Ruskin University, Cambridge CB1 1PT, UK

**Keywords:** protein, AMPK, PGC-1α, substrate oxidation, dietary intake, endurance performance, runners

## Abstract

Manipulating dietary macronutrient intake may modulate adaptive responses to exercise, and improve endurance performance. However, there is controversy as to the impact of short-term dietary modification on athletic performance. In a parallel-groups, repeated measures study, 16 trained endurance runners (maximal oxygen uptake ($\dot{V}$O_2max_): 64.2 ± 5.6 mL·kg^−1^·min^−1^) were randomly assigned to, and provided with, either a high-protein, reduced-carbohydrate (PRO) or a high-carbohydrate (CHO) isocaloric-matched diet. Participants maintained their training load over 21-consecutive days with dietary intake consisting of 7-days habitual intake (T1), 7-days intervention diet (T2) and 7-days return to habitual intake (T3). Following each 7-day dietary period (T1–T3), a micro-muscle biopsy was taken for assessment of gene expression, before participants underwent laboratory assessment of a 10 km treadmill run at 75% $\dot{V}$O_2max_, followed by a 95% $\dot{V}$O_2max_ time to exhaustion (TTE) trial. The PRO diet resulted in a modest change (1.37-fold increase, *p* = 0.016) in AMPK expression, coupled with a significant increase in fat oxidation (0.29 ± 0.05 to 0.59 ± 0.05 g·min^−1^, *p* < 0.0001). However, a significant reduction of 23.3% (*p* = 0.0003) in TTE post intervention was observed; this reverted back to pre levels following a return to the habitual diet. In the CHO group, whilst no change in sub-maximal fuel utilisation occurred at T2, a significant 6.5% increase in TTE performance (*p* = 0.05), and a modest, but significant, increase in AMPK (*p* = 0.042) and PPAR (*p* = 0.029) mRNA expression compared to T1 were observed; with AMPK (*p* = 0.011) and PPAR (*p* = 0.044) remaining significantly elevated at T3. In conclusion, a 7-day isocaloric high protein diet significantly compromised high intensity exercise performance in trained runners with no real benefit on gene markers of training adaptation. A significant increase in fat oxidation during submaximal exercise was observed post PRO intervention, but this returned to pre levels once the habitual diet was re-introduced, suggesting that the response was driven via fuel availability rather than cellular adaptation. A short-term high protein, low carbohydrate diet in combination with endurance training is not preferential for endurance running performance.

## 1. Introduction

A number of factors can influence endurance performance, including body mass, aerobic and anaerobic capacity, running efficiency, fuel utilisation and energy metabolism [1]. The manipulation of dietary intake has become a popular intervention to target a number of these determinates. A critical component to these factors is mitochondrial function; with mitochondrial efficiency, density and abundance being linked with improved aerobic capacity, exercise efficiency and energy metabolism [2]. Mitochondria are regulated by a number of mechanisms including fission, fusion, autophagy and biogenesis. Puigserver et al. [3] first isolated and identified peroxisome proliferator-activated receptor gamma coactivator 1-alpha (PGC-1α) as having a key functional role in the regulation of mitochondrial processes. It has since been established that a series of transcriptional genes play an essential role in the activation of mitochondrial biogenesis and fatty acid oxidation including: 5′ adenosine monophosphate-activated protein kinase (AMPK), silent information regulator (SIRT1), peroxisome proliferator-activated receptors (PPAR) and PGC-1α [4,5,6]. As such, nutritional interventions (such as increasing energy expenditure [7,8] and manipulating carbohydrate availability [9]), targeting these transcriptional genes have been developed to improve mitochondrial function.

A substantial body of evidence has demonstrated that SIRT1 and AMPK both respond to increases in energy expenditure [7,8] and reduced carbohydrate availability [9]. In addition, it has been suggested that increased dietary carbohydrate intake prevents the activation of the AMPK-SIRT1 axis in skeletal muscle, potentially down-regulating the metabolic pathway, which may blunt acute adaptations to exercise [4,10,11]. Endurance exercise is well established as a potent stimulator of PGC-1α [12,13] both independently and combined with exercising in a low glycogen state [14]. Restricting dietary carbohydrate intake (or training in an acutely depleted state, the ‘train-low, perform-high’ approach) to enhance the adaptation from physical training has been proposed as an effective short-term strategy to enhance metabolic and cellular adaptations associated with enhanced oxidative phosphorylation [13,14,15,16,17]. Such transcriptional changes may trigger mitochondrial biogenesis, increasing the oxidative capacity of the muscle, enhancing beta-oxidation and ultimately sparing muscle glycogen, all of which could lead to enhanced endurance performance. It has been demonstrated that such high fat, low carbohydrate interventions can substantially increase fat oxidation during prolonged cycling [16]. However, high intensity exercise performance subsequent to such dietary manipulation is unlikely to be enhanced, and may even be compromised [16,18,19,20] in the absence of glycogen restoration. Recently, Impy et al. [21] proposed a ‘glycogen threshold hypothesis’, indicating that an optimal glycogen level may be required to optimise adaptive responses to endurance exercise. However, understanding the glycogen cost of habitual training is challenging and a limiting factor in prescribing optimal glycogen-periodisation strategies.

Interventions to reduce carbohydrate intake in endurance athletes tend to substitute in dietary fat to maintain adequate energy intake. However, adherence to such diets for moderate periods of time >5-days diets can be poorly tolerated [22]. An alternative approach to maintain energy intake, but reduce carbohydrate consumption is to substitute dietary carbohydrate for protein. Under resting conditions in young healthy males, a high-protein, low-carbohydrate, hypocaloric diet has been shown to significantly increase AMPK, PPAR and PGC-1α expression [23]. Similar findings have been observed in obese individuals fed a high-fat, low-carbohydrate hypocaloric diet [10]. This suggests that under acute metabolic stress (e.g., caloric restriction or carbohydrate availability) preferential adaptations in cellular metabolic processes may occur subsequent to acute dietary intake modifications [6]. This also infers that different interventions delivering an external stress can be utilised to initiate beneficial metabolic adaptations. One such method to elicit additional external stress alongside endurance training may be by increasing dietary protein as a substitute for carbohydrate; however, there is a paucity of literature describing the response to this. It is also currently unknown how long adaptations to acute metabolic stress may persist, and the impact of removing acute dietary stress has on such adaptations. 

Therefore, the aim of this study was to investigate the impact of a highly controlled short-term high-protein, reduced-carbohydrate (PRO) or a high-carbohydrate (CHO) diet in combination with endurance training on transcriptional biomarkers, fuel utilisation and exercise performance in trained male runners. It was hypothesised that PRO diet in combination with endurance training would increase the expression of transcriptomic markers of mitochondrial biogenesis, which would be sustained following return to habitual dietary intake. It was also hypothesised that no difference in high intensity endurance performance would be observed after the intervention diets, but upon return to habitual dietary intake the PRO intervention would demonstrate an improved performance due to maintained adaptation and restored glycogen availability.

## 2. Materials and Methods

### 2.1. Participants

This study was conducted in accordance with the Declaration of Helsinki (2013), and the protocol was approved by the University of Hertfordshire Life and Medical Sciences Ethics Committee (protocol number LMS/PGR/UH/02227). Following a study briefing and suitability interview, participants were invited to take part. Power calculation assessment for sample size (G *power3, Dusseldorf) using α = 0.05; 1 − β = 0.80; based on observed data of PGC-1α mRNA expression [9] indicated that a total of 6 participants were required per group. All participants were required to be male and between the age of 18–45 years, and were recruited through local running clubs, and research team network. From an initial pool of twenty individuals, three withdrawals were unrelated to the trial, and one did not complete the second trial and was excluded from subsequent analyses. Therefore, sixteen healthy, trained male volunteers completed this study (participant characteristics are shown in Table 1 according to allocated test group). Written informed consent was obtained from all participants included in the study. All participants were required to be free from any medical condition which would conflict with the study parameters. Additionally, all participants were required to refrain from following a restrictive dietary regime, not suffer from food allergies or intolerances, and be prepared to consume a controlled, prescribed dietary intake for each trial period. For training suitability, all participants needed to have completed 10-km in sub-38 min or 5-km in sub-18 min in the previous 12 month period, with a running history >2 years, training ≥4 times per week with no significant change in training load or body mass in the previous 3 months.

### 2.2. Experimental Design and Procedures

In an investigator-blinded, parallel-groups, repeated-measures design, participants were randomly assigned to either: PRO (n = 8; total energy intake macronutrient distribution of 40% protein, 30% carbohydrate, and 30% fat) or energy-matched CHO (n = 8; macronutrient distribution of 10% protein, 60% carbohydrate, and 30% fat). Participants were not informed of group allocation; however, due to the nature of dietary intervention studies it was not possible to blind food items. Participants attended the Human Performance Laboratory (GlaxoSmithKline, Brentford, UK) for baseline testing and a familiarisation session, followed by three experimental sessions (T1, T2, T3). Participants followed their habitual dietary intake for 7-days prior to completing T1, after which they followed their prescribed intervention diet (PRO or CHO) for 7-days prior to T2, and finally returned back to their habitual dietary intake for a further 7-days prior to T3. Participants were requested to complete the same training sessions each week throughout the duration of the study and rest the day prior to each test visit. All experimental procedures were completed in the same order and time of the day.

Baseline and Familiarisation Session: All testing was completed in a temperature- controlled room (TISs Sport Science environmental chamber) set at 20 °C and 40% humidity (averaging 20.7 ± 0.8 °C, 41.3 ± 5.0% humidity and 1010.0 ± 13.6 mmHg air pressure across the trials) using the same treadmill (Cosmed T170, Cosmed, Rome, Italy), which was not moved throughout the experimental period. Fan cooling was provided throughout all testing, with a floor fan positioned 50 cm in front of the treadmill at a 45° angle and set to an air speed of ~5.8 m·s^−1^. Prior to assessment of maximal oxygen consumption ($\dot{V}$O_2max_), participants rested for 5-min, after which height (mechanical stadiometer, Seca 216, seca Ltd., Hamburg, Germany) and weight (digital column scale, Seca 704) were recorded and a heart rate (HR) belt fitted (Polar T31 Coded, Polar Electro Ltd., Warwick, UK). A 20 µL resting, capillarised, fingertip blood sample was then collected, and participants then undertook a standardised 5 min warm up at a running speed of 9 km·h^−1^.

The protocol for the assessment of $\dot{V}$O_2max_ was the same for all participants. A breath-by-breath online gas analyser (Metalyzer 3B, Cortex, Leipzig, Germany) was used to capture continuous respiratory measures. Criteria for the attainment of $\dot{V}$O_2max_ complied with that outlined by the British Association of Sport and Exercise Sciences (BASES—[24]). Following a 20 min recovery period, participants completed a 5 km steady state (SS) familiarisation treadmill run at 70% v$\dot{V}$O_2max_ (~75% $\dot{V}$O_2max_) in a similar manner to the 10 km SS exercise trial detailed below. Once completed, participants had 5 min recovery before completing a maximal sustainable effort at 95% $\dot{V}$O_2max_ (calculated from the previous $\dot{V}$O_2max_ test) following the protocol detailed below. Prior to departure, participants were familiarised with all other testing procedures, and provided with a food diary and a GPS watch (Garmin Vivo Active, Garmin Ltd., Schaffhausen, Switzerland) to log dietary intake and training volume.

Assessment of Habitual Dietary Intake: A 3-day food diary based on estimated household measures was used [25] to collect habitual dietary intake prior to T1 and T3. During the study briefing, the participants were instructed how to complete the food diary and were provided with a comprehensive example. The importance of accuracy and detail was emphasised (e.g., item weight, portion size, meal breakdown, and fluid intake) as was the importance of maintaining current dietary habits and documenting all food and drink consumed. Participants were requested to record dietary intake on 2 weekdays and 1 weekend day in the 7-day period preceding T1 and T3 to estimate habitual intake. All food diaries were analysed by the same researcher using Nutritics Professional Dietary Analysis software (Nutritics Ltd., Co., Dublin, Ireland).

Assessment of Training Volume: It was important that any observed changes were due to the intervention diet and not due to change in training volume. Therefore, participants were requested to maintain the same weekly training programme throughout the study and were requested to replicate, as closely as possible, each training session on the same day and time each week, logging all training sessions with the GPS watch provided. The training sessions were automatically uploaded online (Garmin Connect, Garmin Ltd., Schaffhausen, Switzerland) for the investigative team.

Caloric Intake and Intervention Dietary Prescription: Individual caloric intake for the intervention diet was prescribed to match energy expenditure, and was calculated using basal metabolic rate (BMR) multiplied by a physical activity factor (PAF). The equation of Henry [26] was used to estimate BMR. The PAF was quantified based on occupational and exercise level factors using a modified Harris and Benedict [27] equation, incorporating estimated non-exercise activity thermogenesis.

A total of 34 diet plans (17 CHO, 17 PRO) were formulated by the investigative team, starting at 1800 kcal·d^−^^1^ and increasing by 150 kcal·d^−^^1^ for each plan up to 4200 kcal·d^−^^1^. Participants were assigned the diet closest to their calculated interventional energy intake. The greatest difference between estimated intervention diet and prescribed diet was 75 kcal per day. All meals were produced by Soulmatefood^®^ (Waterfoot, Lancashire, UK) and delivered to each individual participant in two batches (a 3-day then a 4-day supply). Daily food packages consisted of 5 pre-packaged/cooked meals (breakfast, morning snack, lunch, afternoon snack and dinner). Matching energy intake to energy expenditure is challenging [25] and it was important that the participants were not in a negative energy balance for this study. Therefore, in addition to the calculated dietary intake, a further 500 kcal·day^−^^1^ macronutrient-matched meal was provided. Included with the delivery was a daily menu with consumption instructions and participants were briefed that the additional meal was only to be consumed if still hungry after consuming the standard diet and were required to document if the additional meal was consumed. Participants were requested to remain hydrated through the day, only consume foods provided and requested to drink water ad libitum or drinks free from caffeine or additional energy. The mean dietary intake for both prescribed diets is shown in Table 2.

Experimental Laboratory Sessions (T1–T3): After a rest day from exercise and following a standardised pre-testing protocol, participants arrived at the laboratory at the same time of day for each visit. Participants were advised to arrive at the laboratory using the same mode of transport and requested to refrain from morning physical exertion. Upon arrival, body composition assessment (dual energy x-ray absorptiometry (DXA) scan) was undertaken, followed by a resting micro muscle-biopsy. Participants then completed a 10-km treadmill run at 75% $\dot{V}$O_2max_, had 5-min rest, then completed a time to exhaustion (TTE) running trial at 95% $\dot{V}$O_2max_.

Body Composition Assessment: Following measurement of fasted body mass (BM), participants were requested to remove any metal piercings prior to having a full body DXA scan (GE Lunar iDXA, GE Healthcare, Bucks, UK). The DXA scan was performed in accordance with manufacturer’s guidelines for patient positioning and analysed using enCORE Software, version 14.10 (GE Healthcare, Bucks, UK). All scans were performed by the same operator. Results were analysed for fat mass (FM) and fat free mass (FFM).

Micro Muscle-Biopsy: Muscle biopsies were obtained from the midpoint on the lateral aspect of the right vastus lateralis muscle. The biopsy site was cleaned using Betadine (Pardue Products, Stamford, CT, USA) and samples were obtained under local anaesthesia (2 mL of 1% *v/v* without adrenaline, Lidocaine Hydrochloride (Hameln Pharmaceuticals: cat. no PL01502) injected into the subcutaneous fat of the selected biopsy site). Following insertion of a 14-gauge co-axial needle ~2 cm into the muscle, a disposable core biopsy instrument (TSK Stericut Biopsy Needle 14 Gauge, TSK Laboratories, Tochigi-Shi, Tochigi-Ken, Japan) was inserted through the co-axial and activated. A single biopsy pass was used collecting ~15–20 mg of muscle tissue. The biopsy instrument was immediately removed, and within 10 s the muscle tissue was removed from the biopsy instrument using a sterile scalpel and flash frozen in liquid nitrogen. The muscle sample was placed in an individual vial and stored at −80 °C until analysis (HTA license number 12202).

10-km Steady State Treadmill Run: Following a resting 20 µL capillary blood sample, participants completed a standardised 5 min warm up at 10 km·h^−1^ on the treadmill, followed by self-directed stretching. Along with a HR monitor, participants were fitted with an oro-nasal facemask in the same manner as the $\dot{V}$O_2max_ test, connected to an online gas analyser for breath-to-breath assessment of respiratory measures. The steady state 10 km run was calculated to be performed at 75% $\dot{V}$O_2max_, with a rolling start (calculations post-trial indicated an average of 75.6 ± 2.6% $\dot{V}$O_2max_ with no difference between groups observed: PRO = 76.4 ± 3.2%; CHO = 74.7 ± 2.4%, *p* > 0.05). After each kilometre, standardised prompts were provided to participants along with collection of a capillary blood sample and assessment of HR and rated perceived exertion [28]. Rates of carbohydrate and fat oxidation were calculated with the attained $\dot{V}$O_2_ and $\dot{V}$CO_2_ data using stoichiometric equations [29], with protein oxidation assumed negligible.

Time to Exhaustion (TTE) Trial: Following a 5 min recovery period, participants then performed a maximal sustainable effort (MaxSE) test at a speed equivalent to 95% $\dot{V}$O_2max_. No visual or audible encouragement was permitted throughout the trial. Participants ran until volitional fatigue, upon which a final capillary blood sample was collected. The same investigator timed all TTE efforts throughout the study.

### 2.3. Quantitative PCR (qPCR) Preparation and Analysis

RNA Isolation: Human muscle biopsies were homogenised in 700 µL MagNA Pure LC RNA Isolation Tissue Lysis buffer (Roche, Mannheim, Germany) in Roche MagNA Lyser Green bead tubes at 6500 rpm for 30 s. After homogenisation, the tubes were centrifuged for 10 min at 13,000× *g* before 350 µL of each homogenate was used for RNA extraction. Total RNA was extracted using the MagNA Pure 96 Cellular RNA Large Volume Kit on a MagNA Pure 96 (Roche, Mannheim, Germany), in an elution volume of 50 µL according to the manufacturer’s protocol. RNA was stored at −80 °C. RNA concentrations were determined (A260) using a NanoQuant plate on a Tecan Infinite 200PRO. RNA quality (RNA integrity number equivalent, RINe) was assessed using RNA ScreenTape on a 2200 TapeStation (Agilent, Santa Clara, CA, USA).

Reverse Transcription (RT): As a result of the large sample number and the automated process, RNA concentration was not taken into account for each individual reaction. Fourteen µL of each RNA sample was reverse transcribed in a total volume of 20 µL using the High capacity cDNA reverse transcription kit (without RNase inhibitor, Applied Biosystems, Thermo Fisher, Loughborough, UK). The average RNA concentration across the 142 samples was 32.4 ng·µL^−1^ equating to an average of 453 ng RNA/reverse transcription reaction. Reactions were performed in 96 well PCR plates on a PTC-225 Peltier thermal cycler (MJ Research, St. Bruno, Quebec, Canada) using the following profile: 25 °C for 10-min, 37 °C for 60-min, 85 °C for 5-min, 4 °C hold. Minus RT control reactions were set up for 14 samples, in which the RNA component was replaced with nuclease free water (Ambion, Fisher Scientific, Loughborough, UK).

qPCR Analysis: For quantitative real-time PCR (qPCR), Human TaqMan^®^ gene expression assays were purchased as a 20× assay ready stock from Life Technologies (Carlsbad, USA) (primers 18 mM and probes 5 mM) (Table 3). One µL cDNA was added to each qPCR reaction mixture which also contained gene expression assay mix (primers 900 nM final, probe 250 nM final), LightCycler 480 Probes Master and nuclease free water to give a 5 µL total reaction volume. Reactions were prepared in white multiwell 384 plates (Roche, Mannheim, German) using a Mosquito HV (TTP Labtech, Melbourn, UK). The plates were sealed using optical seals (Roche, Mannheim, Germany) and centrifuged at 290× *g* for 2 min before being run on a LightCycler^®^ 480 instrument (Roche, Mannheim, Germany) with the following thermal cycling parameters: initial de-naturation step 95 °C for 10 min, followed by 45 cycles of denaturation at 95 °C for 10 s and primer annealing/extension at 60 °C for 30 s. A cooling step at 40 °C for 30 s was the final stage of the run. The crossing point (Cp) value for each sample was calculated using the second derivative maximum method applied directly by the Roche software to the real-time amplification curves. This value represents the cycle at which the increase of fluorescence is highest and where the logarithmic phase of a PCR begins.

Amplification Efficiency of qPCR Assays: The efficiency of each of the Taqman^®^ assays on demand was confirmed by performing standard curves in a 384 well qPCR assay on the LightCycler^®^ 480 under the same conditions described above. Six point standard curves with 1 in 10 serial dilutions were prepared in nuclease free water for human plasmid DNA for each of the genes being tested alongside Human genomic DNA with a top concentration of 3 × 10^5^ copies/µL and 1 × 10^5^ copies/µL, respectively. qPCR reactions were performed in triplicate and the amplification efficiency calculated for each Taqman^®^ assay on the basis of the equation E = (10 − 1/slope − 1) × 100 with the logarithm of the template concentration on the x axis and the average Cp plotted on the y axis.

Data Analysis of the qPCR Assays: The muscle biopsy qPCR raw Cp values were exported and normalised to the housekeeper gene (GAPDH) using an analysis of covariance method. Relative fold change in expression levels were calculated using the 2-ΔΔCT method.

### 2.4. Statistical Analysis

Statistical analysis was conducted using the statistical package for the Social Science software program (SPSS; version 22, IBM, Armonk, NY, USA). Dependent variable distributions were assessed for Kolmogorov–Smirnov test of normality, along with visual inspection of histograms and boxplots. Mauchly’s sphericity test was used to assess the assumption of sphericity within repeated-measures effects. Unless stated, Mauchly’s test was not significant, therefore the assumption of sphericity was accepted. Group and time point effects were assessed using a two factorial mixed design ANOVA (4 groups, 2 time points) with dietary intake (between) and time point (within) as the main variables. To determine the location of the time-point differences and group interactions, a Bonferroni post hoc test was used. Statistical significance was set at *p* ≤ 0.05. Data presented as mean ± SEMs (unless stated).

## 3. Results

### 3.1. Dietary Intake, Body Composition and Training Data

No significant group interactions or time point differences were observed in habitual dietary intake, body mass, lean mass or fat mass (*p* > 0.05), demonstrating energy intake matched energy requirement during both the dietary intervention and habitual intake weeks. No within- or between-group differences were observed in total training distance covered each week (training distance PRO vs. CHO, *p* = 0.83) across the study period. However, a significant reduction in average running speed was observed between T1 (14.7 km·h^−1^) and T2 (13.8 km·h^−1^) in the PRO group (*p* = 0.047, Table 4). Once reverted back to habitual diet, average training speed for PRO in T3 was 14.2 km·h^−1^.

### 3.2. Transcription Biomarkers

**Figure 1:**
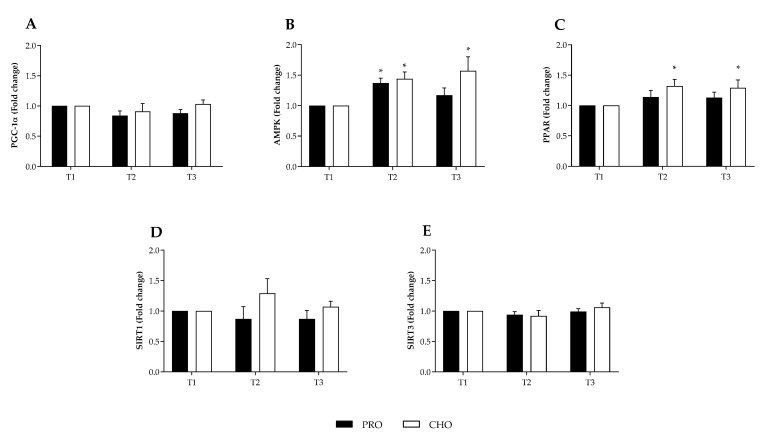
mRNA response (fold change) to habitual and intervention diet phases in combination with endurance training. Habitual dietary intake in first (T1) and third (T3) week. Intervention phase (T2) consisting of either high protein (PRO) or high carbohydrate (CHO) intake. mRNA expression of PGC-1α (**A**), AMPK (**B**), PPAR (**C**), SIRT-1 (**D**), and SIRT-3 (**E**). * Denotes significantly different within group from T1 (*p* < 0.044).

No change was observed in the resting mRNA content of PGC-1α, SIRT1 and SIRT3 at any time point in either group (Figure 1A,D,E). A within group difference main effect (*p* = 0.027) for AMPK mRNA was observed in the CHO group (Figure 1B) and AMPK mRNA expression was significantly elevated at T2 (1.44 fold increase relative to T1, *p* = 0.042), and maintained at T3 (1.57 fold increase relative to T1, *p* = 0.011). A within group difference (*p* = 0.018) was also observed in the PRO group where AMPK mRNA expression was significantly increased at T2 (1.37 fold increase relative to T1, *p* = 0.016); however, this returned to pre-intervention levels by T3 (*p* = 0.18). PPAR expression in the CHO group exhibited the same trend as AMPK, with a 1.32 and 1.29 fold increase at T2 (*p* = 0.029) and T3 (*p* = 0.044) relative to T1, respectively. No time point difference in PPAR expression was observed in the PRO group. No changes were observed for SIRT1 or SIRT3 mRNA expression within or between groups (*p* > 0.05).

### 3.3. Steady State Fuel Utilisation and Related Measures

**Figure 2:**
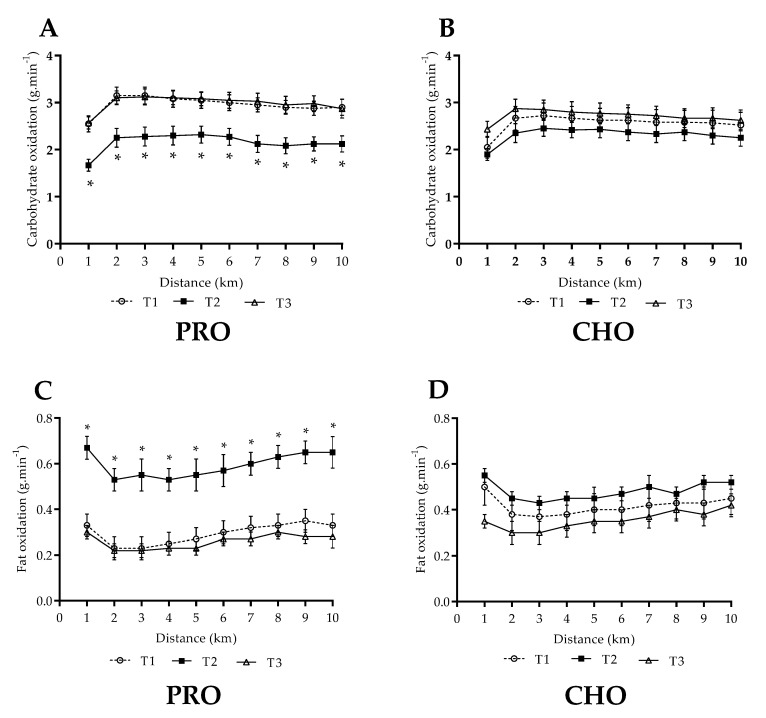
Substrate oxidation rates (g.min^−1^) during a 10-km steady state (~75% $\dot{V}$O_2max_) treadmill run following habitual and intervention diet phases. Habitual dietary intake in first (T1) and third (T3) week. Intervention phase (T2) consisting of either high protein (PRO) or high carbohydrate (CHO) intake. Carbohydrate oxidation (g·min^−1^) in PRO (**A**), carbohydrate oxidation in CHO (**B**), fat oxidation in PRO (**C**) and fat oxidation in CHO (**D**). * Denotes significant difference compared to both T1 and T3 (*p* < 0.05).

In the CHO group, both carbohydrate and fat oxidation remained similar across all trials during the 10-km SS trial (*p* > 0.05, Figure 2B,D). In the PRO group a significant time point difference in fat (*p* < 0.001) and carbohydrate oxidation (*p* < 0.001) was observed, where fat oxidation was significantly increased and carbohydrate oxidation was significantly decreased at each km (1–10 km) collection point in TP2 compared to TP1 (Figure 2A,C, *p* < 0.0001). Mean fat oxidation following PRO increased from 0.29 ± 0.05 to 0.59 ± 0.05 g·min^−1^ (*p* < 0.0001), an increase of 101% relative to T1. Correspondingly, mean carbohydrate oxidation following PRO decreased by 27% from 2.96 ± 0.18 to 2.15 ± 0.19 g·min^−1^ (*p* < 0.0001). Both fat and carbohydrate oxidation returned to pre-intervention levels once reverted back to habitual dietary intake.

**Figure 3:**
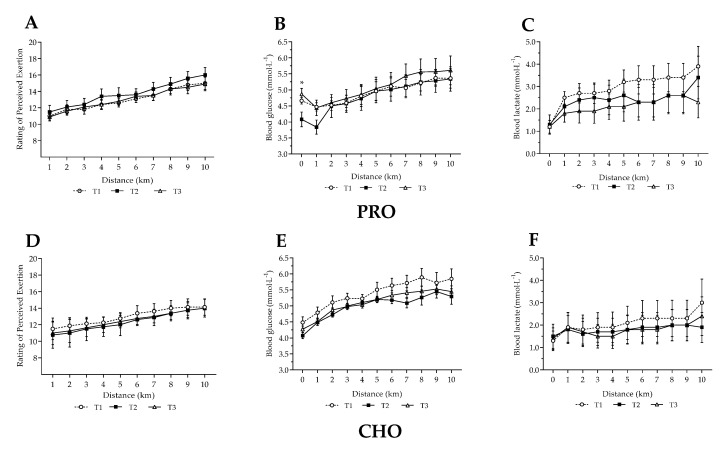
Measures of perceived exertion, blood glucose and lactate during a 10 km treadmill run at 75% $\dot{V}$O_2max_ following habitual and intervention diets. Habitual dietary intake in first (T1) and third (T3) week. Intervention phase (T2) consisting of either high protein (PRO) or high carbohydrate (CHO) intake. All diets undertaken in combination with endurance training. Top graphs (**A**–**C**) refer to PRO intervention, lower graphs (**D**–**F**) refer to CHO intervention.* = T2 significantly different from T1 and T3 within-group (*p* = 0.031).

No differences were observed between trials during the 10 km SS trial in the CHO group for perceived exertion, blood glucose or lactate (Figure 3A–E). A between group difference in blood glucose at rest was observed in the PRO group (*p* = 0.031), with a significant difference observed between the intervention diet and T3 (4.1 ± 0.6 and 4.9 ± 0.7 mmol·L^−1^, *p* = 0.034), and a trend to difference between T1 and T2 (4.7 ± 0.4 vs. 4.1 ± 0.6 mmol·L^−1^, *p* = 0.055). No other differences were observed for PRO, or between groups.

### 3.4. MaxSE Time Trial Results

**Figure 4:**
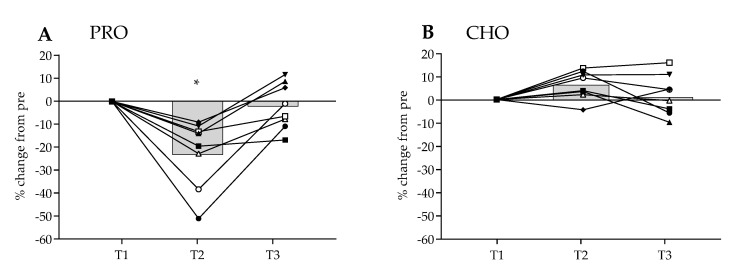
Mean (bar) and individual (line) relative change (%) from pre-intervention maximal sustainable effort test. Time trial to exhaustion (TTE) from a 95% MaxSE trial. Habitual dietary intake in first (T1) and third (T3) week. Intervention phase (T2) consisting of either: (**A**) high protein (PRO) or (**B**) high carbohydrate (CHO) intake. * = significant mean difference (*p* < 0.05) compared to T1.

The results of the 95% MaxSE trial are shown in Figure 4A,B, expressed as relative change (%) from T1. Following PRO, TTE was significantly reduced within group by 23.3% at T2, with all participants exhibiting decreased ability to perform (*p* = 0.0003). Upon subsequent return to habitual dietary intake, TTE for PRO at T3 was comparable to T1 (T1: 128.3 ± 29.3 s, T2: 98.4 ± 31.8 s, T3: 125.3 ± 32.39 s). In contrast, significant (*p* = 0.05) within group change of +6.5% in TTE at T2 was noted in the CHO group with 7 of the 8 participants improving relative to TP1. TTE performance for CHO also returned to pre intervention levels by T3 (T1: 182.2 ± 44.4 s, T2: 194 ± 45.5 s, T3: 184 ± 38.9 s). A between group effect in TTE was observed (*p* = 0.017), with no relative difference reported at T1 or T3, but at T2 there was a significant relative difference in performance (*p* < 0.0001).

## 4. Discussion

In the present study, we determined the impact of a short-term period of either high PRO or CHO dietary intake in trained male runners under controlled conditions on transcription biomarkers of mitochondrial biogenesis, sub-maximal substrate utilisation and high intensity exercise performance. It was hypothesised that a high PRO diet, due to carbohydrate reduction, would positively impact resting transcription biomarkers of mitochondrial biogenesis including an up-regulation of PGC-1α, AMPK and SIRT-1 and such adaptations may be sustained following return to habitual dietary intake. However, the results from the study did not show this, as the gene mRNA expression of the transcriptional biomarkers following the PRO intervention were predominantly insignificant.

Findings from the study demonstrated that following PRO, a significant and profound reduction in high intensity TTE was observed, and this was seen in all participants. The moderate increase in resting AMPK expression observed at T2 corresponded with a significant increase in fat oxidation rates during sub-maximal 10 km exercise. Whilst such changes may have implications for endurance training, AMPK returned to pre-intervention levels upon return to habitual intake, as did substrate utilisation. Thus, a lack of responses in other transcription biomarkers following PRO, as well as a return to pre-intervention performance levels following the second habitual dietary period infer that any beneficial impact of a short-term PRO diet on fuel utilisation may be limited to energy availability. This coupled with the highly significant and inclusive reduction in high intensity running performance suggests that PRO regime is likely not to be preferential for endurance athletes seeking to optimise performance.

By reducing carbohydrate intake below habitual levels, but maintaining energy intake, it was expected that a short-term cellular energy demand would be challenged in favour of an up-regulation of SIRT-1 and AMPK leading to greater expression of PGC-1α at rest. This, however, did not occur, suggesting that during periods of endurance training, athletes may have limited gene transcription benefits from consuming an acute high protein, carbohydrate-reduced diet. In a similar manner to low-carbohydrate high-fat diets, the increase in steady state fat oxidation observed in the PRO group is not unexpected, considering the reduced dietary carbohydrate availability. Thus, different diets which reduce habitual carbohydrate intake below individual thresholds for daily glycogen repletion, may lead to a metabolic ‘shift’ towards increased beta-oxidation to meet energy demands. It is likely such changes in respiratory exchange ratio reflect substrate availability rather than true adaptations within the muscle [14]. Furthermore, it is important to consider the amplitude of change observed in the resting gene expression levels. Post exercise, a >10-fold increase in such genes has been previously observed [13], whereas in the current study we observed <1.5 fold increased in selected gene expression. As limited literature exists reporting resting expression of such genes in training studies the finding of this study provides novel insight into the short term adaptive response. However, with such small, but in some cases significant, changes in gene expression, the interpretation should be viewed with caution and further work is required to fully establish the importance of these findings.

In contrast to a high PRO diet, participants following the CHO condition had a mean increase in both AMPK and PPAR expression (of similar 1.5 fold amplification), which was sustained within-group following return to habitual dietary intake. Despite no changes in sub-maximal substrate utilisation following CHO, a small but significant (6.5%) improvement in high intensity running performance was noted at T2. Furthermore, it was observed that training intensity during CHO was maintained (no difference in weekly average speed). However, following the PRO intervention, these participants exhibited a reduction in mean training intensity (average speed decrease of 0.9 km·h^−1^), which may seem minimal, but a 1 km·hr^−1^ reduction in running speed would equate to a 10 km time approximately 2 min 30 s slower (at the speeds the participants perform at). It is feasible, therefore, that this reduction in training intensity reduced the cellular stress in the PRO group compared to CHO. Indeed, this may explain why transcription biomarkers were sustained at T3 in the CHO group. The inclusion of a high carbohydrate approach (60% of energy intake) may have favoured greater glycogen recovery between training bouts enabling the maintenance on training intensity, which ultimately increases metabolic stress and is likely to be the main mechanism stimulating the observed training adaptation. Previously, it has been demonstrated that increasing exercise intensity induces greater changes in PGC-1α expression [30], suggesting that a reduction in training intensity observed subsequent to the PRO intervention is likely be counterproductive to adaptation. As such, this supports the contention that carbohydrate intake should not be compromised during periods of moderate to high training volume in experienced athletes, seeking high levels of athletic performance.

Carbohydrate availability has been shown to impact endurance exercise [31,32,33]. Increasing carbohydrate availability through either glycogen sparing or through improved efficiency of exogenous provision during sustained exercise has been demonstrated to enhance endurance performance [34,35,36]. An increase in habitual carbohydrate intake to contemporary recommended guidelines (7–12 g·kg^−1^·d^−1^, [31]) for trained athletes observed in the current study resulted in maintenance of training volume/maximal sustained performance. The exercise-induced changes in energy-sensing mechanisms during periods of regular endurance training may provoke sustained adaptations in both AMPK and PPAR mRNA. Moderate to high intensity endurance exercise is known to increase AMPK activity due to metabolic stress and accelerated ATP demand. In the presence of adequate carbohydrate provision, the up-regulation in AMPK and PPAR mRNA observed in the current study may be associated with higher GLUT-4 and hexokinase activity [37] as opposed to mitochondrial biogenesis. As such, the combination of higher carbohydrate intake, coupled with regular endurance training may have favoured adaptations associated with glucose metabolism and sustained carbohydrate oxidation rates. For athletes undergoing demanding or high-volume endurance training where performance maintenance is important, this study indicates that a high CHO approach may be fundamental to this process.

Uniquely, in this study, dietary substitution of high protein (not fat) was employed during the reduced carbohydrate phase. The high PRO intervention significantly impacted fuel utilisation during sustained endurance exercise, with mean peak fat oxidation rates reaching 0.59 g·min^−1^. The authors knowledge that this is the first time such changes following a high PRO approach have been reported. These findings are similar to those previously reported subsequent to a high fat, low carbohydrate diet [16], where mean fat oxidation reached 0.6 g·min^−1^ during sub-maximal cycling. Short term reductions in carbohydrate intake below habitual levels by either increasing dietary fat or protein intake has similar effects on respiratory exchange ratio during sub-maximal exercise, suggesting that it is carbohydrate availability, rather than the increase in either dietary fat or protein, which dictates fuel utilisation during sub-maximal exercise. The longer-term use of high fat approaches, leading to short-term fat adaptation [14,38] or the longer-term ‘keto-adaptation’ response [39], compared with longer-term use of high protein phases is warranted to assess metabolic changes during sustained training periods.

Whilst PRO in the current study was found to reduce carbohydrate and increase fat utilisation during a 10 km steady state treadmill run, it is important to remember that high intensity performance was compromised at T2. Furthermore, the return to pre-intervention levels for transcription biomarkers, along with a ‘normalisation’ in fuel efficiency by T3 (habitual intake) indicates that there are limited, if any, performance benefits of consuming a high PRO (reduced carbohydrate) diet for 7-days. Whilst the use of PRO may confer positive benefits in terms of fuel efficiency, such findings should be interpreted with caution. Endurance athletes who are looking at manipulating dietary intake during moderate-high intensity training may be better to adopt other feeding regimes, such as short-term low glycogen availability periods, followed by carbohydrate restoration to minimise both training disruptions and/or performance decrements [21].

### 4.1. Applications

Endurance exercise is a potent stimulator of PGC-1α via the AMPK-SIRT1 pathway [40,41], and increases in PGC-1α expression have been observed following a single bout of endurance exercise [13]. This response can be further amplified when in a single exercise bout in a low glycogen state [42,43]. Furthermore, it has been demonstrated in vitro [8] and in vivo [10] that reduced glucose and/or nutrient availability promote increased PGC-1α, AMPK and SIRT1 mRNA expression. In untrained individuals over a 14-day training period, Perry et al. [13] found a transient post-exercise response in PGC-1α expression, observing a 10-fold increase in PGC-1α mRNA expression subsequent to the first bout of endurance cycling, which was reduced to a 4-fold increase after the seventh training session. In addition, with adequate glycogen availability, Psilander et al. [43] found a 2.5-fold increase in PGC-1α mRNA expression following a single bout of endurance cycling in well-trained cyclists ($\dot{V}$O_2max_ = 65.4 ± 0.9 mL·kg^−1^·min^−1^), and with the addition of glycogen restriction to the endurance session, an 8.1-fold increase in expression; similar to that observed in untrained individuals [13]. These data sets suggest that the transcriptional adaptive response is blunted when skeletal muscle becomes accustomed to the exercise stress, and increasing the metabolic stress by restricting glycogen availability may result in enhanced post exercise transcriptional adaptation to a single one-off exercise bout. However, these studies examine the impact of carbohydrate restriction after a single bout of exercise. The results from the current study provide a different perspective on the adaptive response and performance outcomes using such nutrient manipulation models, as carbohydrate restriction was prolonged for 7 days. The data does not support the use of repeated days of carbohydrate restriction on training adaptation and exercise performance, demonstrating that reduced carbohydrate availability, without repletion, during a typical training week resulted in a significant decrease in exercise performance and no discernible improvement in resting PGC-1α mRNA expression. Thus, it is likely the PRO intervention utilised in this trial is not a positive method to implement to improve endurance performance.

### 4.2. Study Limitations

An important consideration is that post exercise muscle biopsies were not collected in the current study. However, the results from our previous research [23], along with others [10], demonstrate that mRNA expression is sensitive to dietary manipulation at rest. In addition, Baar et al. [12] found that PGC-1α protein responds similarly to mRNA expression, thus an assumption can be made that an increase in mRNA expression is accompanied by increased protein content. Additionally, despite initial power calculation, the effect size of the mRNA expression is fairly small, thus a larger sample size may be warranted to further validate these findings. As such these aspects of the results should be interpreted with due consideration.

Furthermore, muscle glycogen was not measured, however seminal work from Bergstrom et al. [44] demonstrated that consuming a very low carbohydrate diet for 7-days without exercising resulted in a 32% reduction in muscle glycogen. Thus, it is fair to assume that the high protein intervention, in combination with endurance training, resulted in significant reductions in muscle glycogen. The significant reduction in resting blood glucose, and increase in fat oxidation, during the sub-maximal trial in the high protein group corroborate this further.

## 5. Conclusions

In trained male runners and in combination with habitual training, a 7-day high protein diet significantly increases fat oxidation during sub-maximal exercise, but significantly compromised training intensity and high-intensity running performance. In contrast, training intensity was not impacted subsequent to a 7-day high carbohydrate diet, and neither was sub-maximal substrate utilisation, but an improvement in high-intensity running performance was observed. The high carbohydrate diet also resulted in greater changes in resting muscle AMPK and PPAR expression, which remained elevated on return to habitual diet. These findings suggest that consuming a high protein, low carbohydrate diet for 7-days results in decreased exercise performance and does not enhance training adaptation, thus is not a preferred intervention for endurance athletes to adopt.

## Figures and Tables

**Table 1:** Baseline participant characteristics according to allocated starting group (mean ± SD).

Group	Age (years)	Height (cm)	Body Mass (kg)	$\mathrm{Absolute}\;\dot{V}$O_2max_ (L·Min^−1^)	Relative $\dot{V}$O_2max_ (mL·kg^−1^·Min^−1^)
PRO (n = 8)	25 ± 4	179.4 ± 6.4	69.5 ± 3.3	4.38 ± 0.35	63.1 ± 4.8
CHO (n = 8)	27 ± 5	181.6 ± 3.5	67.6 ± 6.1	4.39 ± 0.28	65.3 ± 6.4

PRO: high protein diet; CHO: high carbohydrate diet. $\dot{V}$O_2max_: maximal oxygen uptake. No differences observed between groups (*p* > 0.05).

**Table 2:** Calculated mean dietary intake and macro-nutrient breakdown for each group during the 7-day intervention.

Group	Energy Intake		Carbohydrate		Protein		Fat	
	kcal·d^−1^	kcal·kg^−1^·d^−1^	g·d^−1^	g·kg^−1^·d^−1^	g·d^−1^	g·kg^−1^·d^−1^	g·d^−1^	g·kg^−1^·d^−1^
PRO	3185 ± 84	48 ± 1.2	239 ± 6.3	3.4 ± 0.9	319 ± 8.4	4.6 ± 0.1	106 ± 2.8	1.5 ± 0.04
CHO	3281 ± 69	49 ± 1.0	492 ± 10.4 *	7.3 ± 0.1 *	82 ± 1.7 *	1.2 ± 0.03 *	109 ± 2.3	1.5 ± 0.03

Prescribed dietary intake (mean ± S.E.) based on estimated energy requirements. PRO = high protein/reduced carbohydrate diet (40/30/30—protein, carbohydrate, fat ratio); CHO = high carbohydrate/typical protein diet (60/10/30—carbohydrate, protein, fat ratio). * denotes significant difference between groups (*p* ≤ 0.002).

**Table 3:** Taqman Assays on Demand information (Applied Biosystems).

Gene Name	Gene Abbreviation	Assay on Demand Number
Peroxisome proliferator-activated receptor gamma coactivator 1-alpha (PGC-1α)	PPARGC1	Hs01016719_m1
AMP-activated protein kinase 1 (AMPK)	PRKAA1	Hs01562315_m1
Peroxisome proliferator-activated receptor delta (PPARδ)	PPARδ	Hs00987011_m1
Silent information regulator-T1 (SIRT1)	SIRT1	Hs01009005_m1
Silent information regulator-T3 (SIRT3)	SIRT3	Hs00953477_m1
Glyceraldehyde 3-phosphate dehydrogenase (GAPDH)	GAPDH	Hs99999905_m1

**Table 4:** Running distance and speed during each week of training throughout the duration of the study.

	T1		T2		T3	
	Distance (km)	Average Pace (km·h^−1^)	Distance (km)	Average Pace (km·h^−1^)	Distance (km)	Average Pace (km·h^−1^)
**PRO**	51 ± 26	14.7 ± 1.7	51 ± 28	13.8 ± 1.5 *	50 ± 28	14.2 ± 1.3
**CHO**	62 ± 20	13.9 ± 0.8	64 ± 24	14.1 ± 1.4	65 ± 22	13.9 ± 0.9

* Denotes significant difference to T1 within group (*p* = 0.047).

## Data Availability

The data presented in this study are available on request from the corresponding author. The data are not publicly available due to ethical considerations, in accordance with consent provided by participants on the use of confidential data.
